# Supplementary figures and images for: Impact of Insurance Benefits and Education on Point-of-Care Ultrasound Use in a Single Emergency Department: An Interrupted Time Series Analysis
Source: Medicina (Kaunas). 2022 Feb 1;58(2):217. doi: 10.3390/medicina58020217 (PMC8878237; doi:10.3390/medicina58020217)

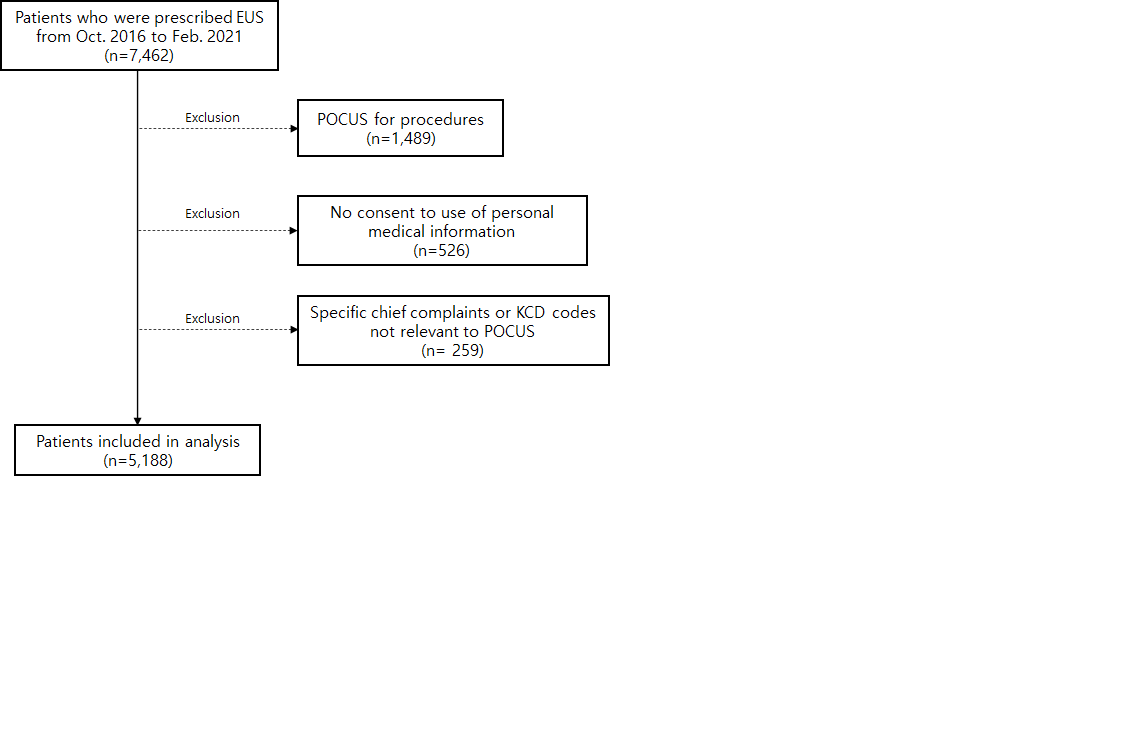

Supplement: Supplementary file 1 [file medicina-58-00217-s001.zip › Figure 1.tif]

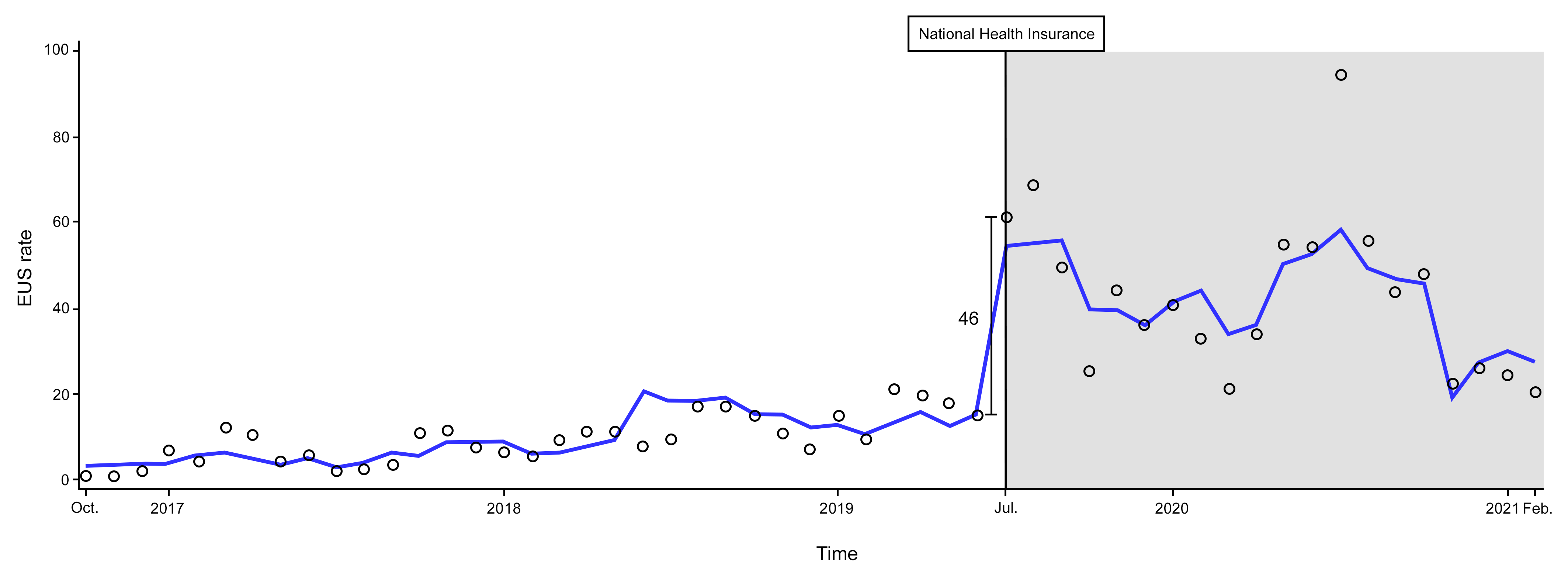

Supplement: Supplementary file 1 [file medicina-58-00217-s001.zip › Figure 2.tif]

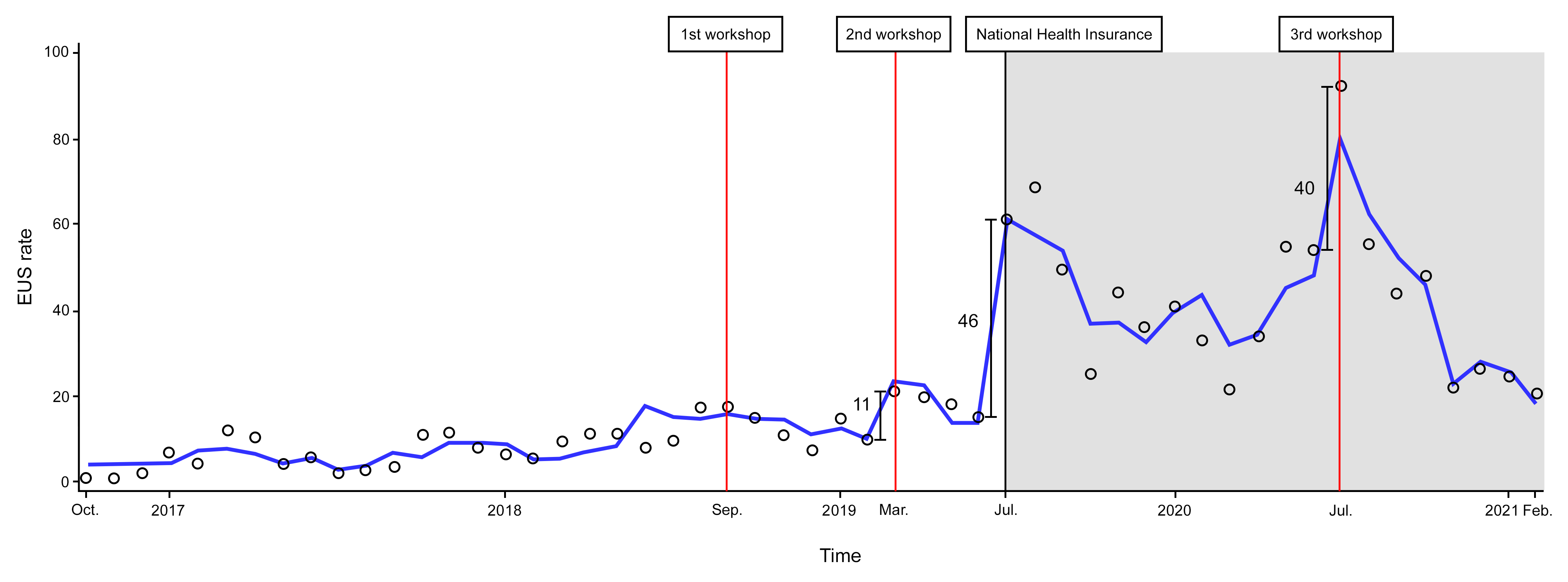

Supplement: Supplementary file 1 [file medicina-58-00217-s001.zip › Figure 3.tif]

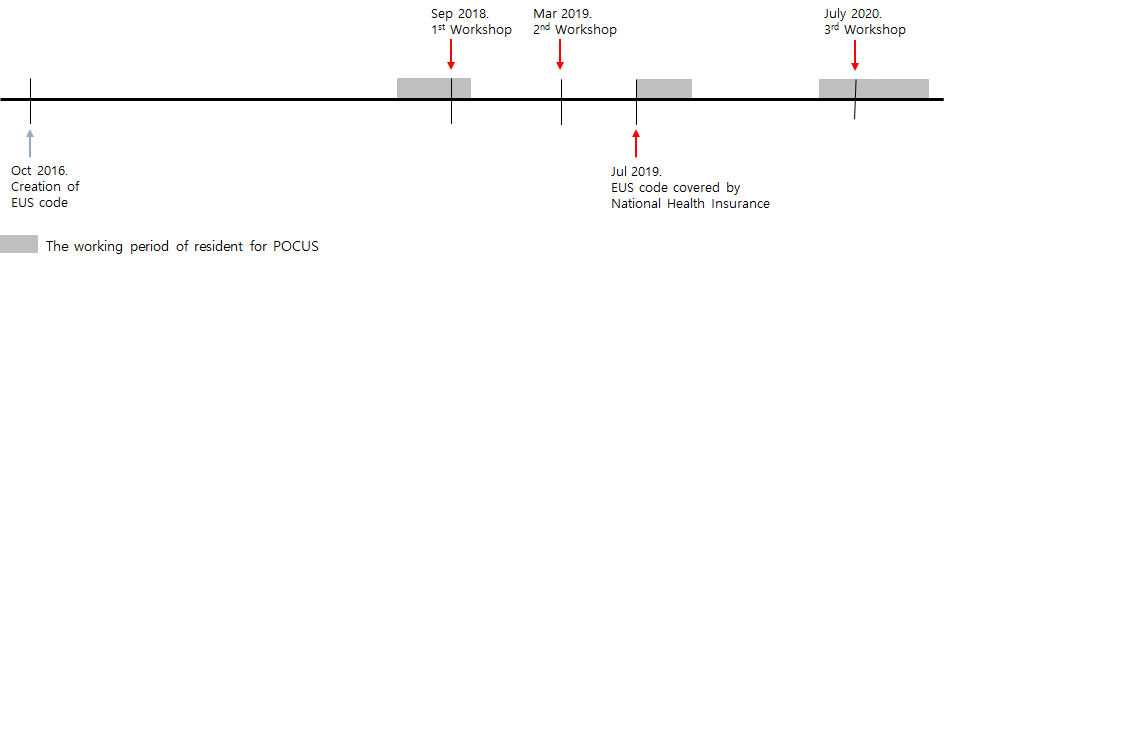

Supplement: Supplementary file 1 [file medicina-58-00217-s001.zip › Figure S1.tif]
